# Detection of Low MAP Shedder Prevalence in Large Free-Stall Dairy Herds by Repeated Testing of Environmental Samples and Pooled Milk Samples

**DOI:** 10.3390/ani12111343

**Published:** 2022-05-25

**Authors:** Annika Wichert, Elisa Kasbohm, Esra Einax, Axel Wehrend, Karsten Donat

**Affiliations:** 1 Animal Health Service, Thuringian Animal Diseases Fund, Victor-Goerttler-Straße 4, 07745 Jena, Germany; awichert@thtsk.de (A.W.); eeinax@thtsk.de (E.E.); 2 Clinic for Obstetrics, Gynaecology and Andrology for Large and Small Animals with Veterinary Ambulance, Justus-Liebig-University Gießen, Frankfurter Straße 106, 35392 Gießen, Germany; axel.wehrend@vetmed.uni-giessen.de; 3 Institute of Mathematics and Computer Science, University of Greifswald, Walther-Rathenau-Straße 47, 17489 Greifswald, Germany; elisa.kasbohm@uni-greifswald.de

**Keywords:** *Mycobacterium avium* subsp. *paratuberculosis* (MAP), environmental samples, milk pools, fecal culture, real-time PCR, ELISA, latent class model

## Abstract

**Simple Summary:**

Paratuberculosis is a disease which affects ruminants worldwide. Many countries have implemented certification and monitoring systems to control the disease, particularly in dairy herds. Monitoring herds certified as paratuberculosis non-suspect is an important component of paratuberculosis herd certification programs. The challenge is to detect the introduction or reintroduction of the infectious agent as early as possible with reasonable efforts but high certainty. In our study, we evaluated different low-cost testing schemes in herds where the share of infected animals was low, resulting in a low within-herd prevalence of animals shedding the bacteria that causes paratuberculosis in their feces. The test methods used were repeated pooled milk samples and fecal samples from the barn environment. Our study showed that numerous repetitions of different samples are necessary to monitor such herds with sufficiently high certainty. In the case of herds with a very low prevalence, our study showed that a combination of different sampling approaches is required.

**Abstract:**

An easy-to-use and affordable surveillance system is crucial for paratuberculosis control. The use of environmental samples and milk pools has been proven to be effective for the detection of *Mycobacterium avium* subsp. *paratuberculosis* (MAP)-infected herds, but not for monitoring dairy herds certified as MAP non-suspect. We aimed to evaluate methods for the repeated testing of large dairy herds with a very low prevalence of MAP shedders, using different sets of environmental samples or pooled milk samples, collected monthly over a period of one year in 36 herds with known MAP shedder prevalence. Environmental samples were analyzed by bacterial culture and fecal PCR, and pools of 25 and 50 individual milk samples were analyzed by ELISA for MAP-specific antibodies. We estimated the cumulative sensitivity and specificity for up to twelve sampling events by adapting a Bayesian latent class model and taking into account the between- and within-test correlation. Our study revealed that at least seven repeated samplings of feces from the barn environment are necessary to achieve a sensitivity of 95% in herds with a within-herd shedder prevalence of at least 2%. The detection of herds with a prevalence of less than 2% is more challenging and, in addition to numerous repetitions, requires a combination of different samples.

## 1. Introduction

Paratuberculosis is a globally prevalent disease caused by an infection with *Mycobacterium avium* subsp. *paratuberculosis* (MAP). Infected cattle suffer from an incurable chronic granulomatous enteritis. The clinical stage of the infection is characterized by aqueous diarrhea with loss of proteins, and ensuing oedema and cachexia [1,2].

Not only for reasons of animal welfare but also for reasons of public health, many countries have implemented paratuberculosis control or herd certification programs [3]. The zoonotic potential of MAP has been widely discussed [4,5,6,7]. Additional reasons for controlling the disease are economic impacts [3] caused by milk loss [8], higher susceptibility to other diseases, increased total losses and culling rates [9], as well as reduced slaughter weights [10].

A systematic review of risk factors associated with the introduction of MAP into dairy herds highlighted the role of purchased animals in between-herd transmission of the disease and, therefore, the importance of herd certification and monitoring systems [11]. Even when tested individually for MAP or MAP-specific antibodies with negative results, purchased animals can be in the subclinical stage of the disease. The interferon gamma response test can be a useful method for detecting early stages of the disease, but the practicability of this test is hampered by its limited specificity, specific pre-analytical requirements, and its high costs [12,13,14]. Testing animals in the subclinical stage with ELISA is not sufficient because of the limited sensitivity of available tests. The informative value of testing an individual animal can be increased by considering (historical) test results of its herd [15,16], as the MAP-status of the herd indicates the risk of the introduction of paratuberculosis by animals of the respective herd [17]. That is why it is important to purchase only animals from herds with low risk of MAP-infection [18,19]. Purchasing only such animals has a considerable effect on the confidence of freedom of MAP non-suspect herds [20].

Hence, there is a need both to identify animals and herds with a low risk of being MAP-infected [17,21], and for the reliable certification of herds. Plenty of research has been conducted regarding this issue, but it remains challenging to detect herds with a very low within-herd prevalence of MAP shedders [22,23,24] with reasonable efforts and at acceptable costs [25]. Detecting a low-prevalence of MAP-shedders in the respective herds with a high certainty is a precondition for monitoring herds that were certified as MAP non-suspect, because these herds are expected to have low number of cases in the first years after the (re)introduction of the infectious agent [19,26].

A study evaluating MAP-surveillance methods revealed that annual bulk milk testing by ELISA was the strategy with the lowest costs [27]. Especially in herds with a low prevalence of MAP shedders, the median herd-level sensitivity of bulk milk testing by ELISA is low (<10%) and the specificity is lower than 100% [27]; the former factor leads to a low detection rate, especially in herds with a low prevalence of MAP shedders [28], and the latter to false positive results. The herd sensitivity of analyzing individual milk samples by ELISA is also influenced by the within-herd-prevalence, and is reduced in herds with a low prevalence [29]. As both the costs and the sampling efforts of (bulk) milk testing are low, sensitivity could be improved by testing herds several times per year [27,30,31].

Another study comparing detection methods for MAP-infected herds found that, regardless of the within-herd prevalence, using environmental samples was the most cost-effective method compared with pooled or individual fecal testing by culture, or serum ELISA followed by fecal culture [32]. In accordance with other studies, this study also showed that the sensitivity of environmental testing depends on the within-herd prevalence [33,34,35] and is not sufficient for the detection of low-prevalence herds [36,37,38,39]. The sensitivity of ten environmental samples is estimated to be about 38% in herds with a prevalence smaller than 5% [32]. Several studies found that repeated collection of environmental samples is an option for increasing sensitivity [40,41], albeit in one study two sampling events resulted in only a minimal increase in sensitivity compared with one sampling event [33]. Another study estimating the within-herd prevalence thresholds for the detection of MAP in dairy herds found no significant reduction in the threshold for repeated environmental samples analyzed by fecal culture and PCR, but repeated sampling increased the probability of detecting a MAP-positive herd [24]. Consequently, there is a long-lasting controversy as to whether repeated environmental sampling yields a sensitivity high enough to be applied in low-prevalence herds or in MAP non-suspect herds that have to be monitored, and more research on this field has been proposed [24,25,34,35].

We hypothesized that repeated testing of dairy herds with environmental samples or pooled milk samples is a sensitive method to monitor herds certified as MAP non-suspect. The objective of our study was to estimate the cumulative sensitivity and specificity for up to twelve sampling events, taking into account the between- and within-test correlation using different test materials (environmental samples, pooled milk samples), combinations of them, and different test methods (fecal culture, real-time PCR, ELISA). Our goal was to identify a testing scheme that achieved a high sensitivity in herds with a low prevalence of MAP shedders, and to identify the number of repeated samplings needed to achieve a sensitivity of at least 95% in such herds.

## 2. Materials and Methods

### 2.1. Study Herds

We conducted a prospective study in 36 large dairy herds housed in free stalls in Thuringia, federal state of Germany. All study herds participated in the voluntary Thuringian paratuberculosis control program, which has been described recently [42,43]. The paratuberculosis status of each herd and the within-herd prevalence of MAP-shedders were known from annual (in the case of MAP-infected herds) or biennial (in the case of herds certified as ‘MAP non-suspect’) individual testing of all lactating cows within a herd by fecal culture or real-time PCR. The mean herd size was 507 cows, with a minimum of 111 and a maximum of 1153 cows. The main breed was German Holstein, with a minor percentage of other breeds (Simmental, Jersey, Brown Swiss) or crossbred cows in several herds. A description of the Thuringian cattle population and the dairy industry has been given elsewhere [43].

Based on the results of the individual fecal samples taken within the context of the Thuringian paratuberculosis control program during the year of the study, the estimated true within-herd prevalence of MAP shedders (i.e., infectious cows) and the 95% confidence interval for each study herd was estimated using the R package epiR [44] with the following inputs:

- Count of positive test results in each study herd;
- Count of individual fecal samples taken in each study herd;
- Imperfect sensitivity of individual fecal culture or real-time PCR: 0.74 [45] (we assumed that fecal PCR is at least as sensitive as fecal culture);
- Specificity of individual fecal culture or real-time PCR: 1.00.

The results of individual fecal testing were used as a reference test regarding herd status. Herds with no MAP-positive individual fecal sample were considered as MAP non-suspect (group 0). All study herds with at least one MAP-positive individual fecal sample were considered as MAP-infected herds. These herds were divided into two groups: One group containing all MAP-infected herds with an estimated true prevalence of less than 2% (group 1) and the other group containing all other MAP-infected herds (group 2). In one study herd, individual fecal samples were not taken as planned during the year of the study, and the results of individual fecal samples taken during the preceding year were used to estimate the true prevalence of this herd.

### 2.2. Environmental Samples

From January 2020 to January 2021, veterinarians from the Animal Health Service visited each study herd twelve times. At each on-farm visit, the veterinarians collected five different samples. The sampling locations were chosen on the basis of studies which found that the environment of lactating cows is more suitable for the detection of MAP than calving or sick cow pens [37,46,47] and that MAP can be detected most frequently in main alleyways and manure storage areas [36,38,48]. Therefore, fecal samples from the barn environment were collected at the following sampling locations:

- milking area (waiting pen);
- main alleyway;
- lactating cow floor (fresh cow pen).

If possible, the collection of feces from single manure piles was avoided. Instead, the veterinarians grabbed mixed feces using a new single-use glove at each sampling site, and put them into sterile plastic cups with a barcode as provided by the laboratory. Additionally, the liquid manure samples and feces taken at the same on-farm visit from the milking area, the main alleyway, and the lactating cow floor were pooled.

When walking around during fecal sample collection, a boot swab sample (“sample sock”) was taken. A single-use boot cover, worn under the sample sock, was used to prevent contamination. In addition, the veterinarian or a member of the farm staff took a sample from the manure pit. After transportation to the laboratory on the same day, the samples were stored at −20 ± 5 °C.

### 2.3. Milk Samples and Milk Pools

For our study, we used individual milk samples taken in the context of the monthly dairy herd improvement test in 2020. Because some herds were not tested each month, samples taken from the respective herds at the beginning of 2021 were used to generate a complete data set comprising twelve milk recordings for each study herd. Individual milk samples dedicated to the same test day and herd were pooled both in groups of 25 and 50 by pipetting 200 µL of each individual sample into a tube. A new pipette tip was used for each sample to prevent carry-over. The composition of each pool was determined by the milking and sampling order. If the number of individual milk samples of one month and herd was not divisible by 25, the remaining samples were pooled and these pools were also handled in the same way as pools of size 25. In addition, pools of size 50 were generated by pipetting 200 µL of two pools of size 25 dedicated to the same herd and test day into one tube. After the pools were generated, they were stored at 5 ± 3 °C until being analyzed by ELISA.

### 2.4. Laboratory Methods

For laboratory analysis, the sample socks were processed as described elsewhere [49]. The sample socks, each environmental sample, and the pool containing the liquid manure and environmental samples were analyzed by both real-time PCR and bacterial culture.

Bacterial culture was conducted according to the accredited method of the official manual of diagnostic procedures of the Friedrich-Loeffler-Institut (FLI), as described previously [49]. Briefly, 3 g of each sample were decontaminated using 0.75% hexadecyl pyridinium chloride solution. After shaking, sedimentation, and decantation of the sample, three slopes of Herrold’s Egg Yolk Medium with Mycobactin (Becton Dickinson GmbH, Heidelberg, Germany) were inoculated with each 200 μL of the sediment. After a one-week incubation in an inclined position at 37 ± 2 °C under microaerobic conditions, the slopes were put upright and incubated at 37 ± 2 °C for an additional 12 weeks. The first semi-quantitative assessment of the slopes was carried out after four weeks of incubation and was repeated every two weeks. Characteristic colonies were differentiated by an IS900 PCR using primers described by Englund et al. [50].

For direct fecal real-time PCR, up to 200 mg of the sample were pipetted into a Precellys Lysis Tube (Bertin Technologies SAS, Montigny-le-Bretonneux, France), together with 1 mL PowerBead Solution (Qiagen GmbH, Hilden, Germany). Using the Precellys 24 homogenizer (Bertin Technologies SAS, Montigny-le-Bretonneux, France), the samples were homogenized three times for 30 s at 6800 rpm and centrifuged at 16,000× *g* for 3 min. Then, 200 µL of the supernatant were used for automatic DNA extraction with the IndiSpin QIAcube HT Pathogen Kit (INDICAL BIOSCIENCE GmbH, Leipzig, Germany) and QIAcube HT (QIAGEN GmbH, Hilden, Germany), according to the manufacturer’s instructions. To detect MAP DNA, the bactotype MAP PCR Kit (INDICAL BIOSCIENCE GmbH, Leipzig, Germany) was applied using the CFX96 TouchTM Real-Time PCR Detection System (Bio-Rad Laboratories GmbH, Feldkirchen, Germany). In addition to the controls included in the kit, a negative internal extraction control was used. Samples with a cycle threshold (Ct) value < 40 were considered to be MAP-positive.

An environmental sample was considered to be MAP-positive if the bacterial culture or the fecal real-time PCR or both produced a positive result; otherwise, the sample was considered to be MAP-negative.

To detect MAP-specific antibodies, all pooled milk samples were analyzed using the commercial ELISA kit ID Screen Paratuberculosis Indirect (ID Vet, Montpellier, France). To avoid loss of sensitivity when using an ELISA kit for analyzing pools instead of individual samples, the cut-off value proposed by the manufacturer should be adapted [51]. The national reference laboratory for Paratuberculosis at the FLI evaluated the use of this test kit for pooled milk samples and recommended a cut-off value of 11% [52]. According to this recommendation, all samples with a sample-to-positive ratio of 11% or higher were considered to be positive. In addition to the positive and negative controls included in the test kit, well-characterized field samples were used as additional positive and negative controls. The results of all pools dedicated to the same herd and the same test day were accumulated to a MAP-positive result if at least one pool had a MAP-positive result; otherwise, the cumulative result for this herd and the respective test day was MAP-negative.

### 2.5. Statistical Analysis and Model

For a combination of the fecal samples from the barn environment (milking area, main alleyway, lactating cow floor), the results were rated as follows: if at least one sample had a MAP-positive result in bacterial culture or PCR, the combination was rated as MAP-positive.

A MAP-negative result was assigned to missing or non-evaluable environmental samples and milk pools. In order to check the influence of this approach on our results, we conducted another calculation after assigning a MAP-positive result to all missing or non-evaluable environmental samples and milk pools.

All in all, we considered 6 binary tests applied at 12 time points in 36 herds. To estimate the sensitivity and specificity at herd level in consideration of between- and within-test correlation, we adapted the Bayesian latent class model for repeated measurements proposed by Wang and Hanson [53]. The likelihood contribution was adapted following the instructions of the authors for applications where the true status (infected or not) is known. Individual fecal testing and the resulting classifications of our study herds (MAP-infected or MAP non-suspect) were considered as the reference test at herd level. It was assumed that the status of a herd is temporally static over the time of examination. Additional model assumptions were constant test sensitivity and specificity over time, as well as exchangeable correlation between repeated applications of the same test on the same herd.

The priors were defined following those proposed by Wang et al., for a hierarchical conditional dependence model [54]. The prior for the temporal covariance was defined as analogous to the prior for the covariances between different tests.

In addition to the estimation of sensitivity, specificity, temporal covariances, and covariances between different tests, the cumulative sensitivity and specificity over time when applying parallel interpretation (positive if any monthly test result is positive) had to be estimated. Therefore, formulae for cumulative sensitivity and specificity, as proposed by Wang and Hanson, were integrated into the model [53]. This approach also allowed for the calculation of the sensitivity and specificity of test combinations. The result of a test combination was specified as positive if at least one test had a positive result (parallel interpretation).

The model was implemented in Just Another Gibbs Sampler (JAGS) [55], based on a related implementation without repeated measurements by Wang et al. [54], and executed in R [56] using the package runjags [57]. Three chains were run in parallel for 100,000 iterations in total, excluding a burn-in period of 5000 samples. The chains started at three different randomly determined initial values and were not thinned. The chains were checked for convergence by visual examination of trace plots and density plots, as well as by means of the potential scale reduction factor (Gelman-Rubin statistic).

The model was run several times with different data as described below. One run was performed with the data of all study herds. Another model run was executed with the data of all MAP non-suspect herds (group 0) and the data of all MAP-infected herds with an estimated within-herd prevalence of less than 2% (group 1). The data of group 0 and all herds with a within-herd prevalence of at least 2% (group 2) were used for a third model run. In contrast to the aforementioned model runs, the fourth model run was performed after assigning a MAP-positive result to all missing or non-evaluable samples (instead of assigning a MAP-negative result to such samples), using the data of all study herds.

Because of the limitations of detecting MAP in fecal samples, we were not absolutely sure about the MAP non-suspect status of all eight MAP-negative study herds. Therefore, we conducted a fifth model run, in which the status of herds with no MAP-positive individual fecal sample was considered to be a latent class, whereas all herds with at least one MAP-positive individual fecal sample were considered to be known MAP-infected herds.

The model code is available at https://github.com/ekasb/lc-repeated-tests (accessed on 27 April 2022).

### 2.6. Comparison of Laboratory Methods

In order to compare the sensitivity of environmental samples analyzed with either bacterial culture or fecal real-time PCR, we conducted two additional model runs. For these runs, the data of all herds were used. The results of individual testing were used as a reference at herd level. For both model runs, the ELISA results (milk pools) were used together with either only the results of fecal culture (environmental samples) or the results of fecal PCR (environmental samples). A MAP-negative result was assigned to all missing or non-evaluable samples.

## 3. Results

### 3.1. Study Herds

**Figure 1:**
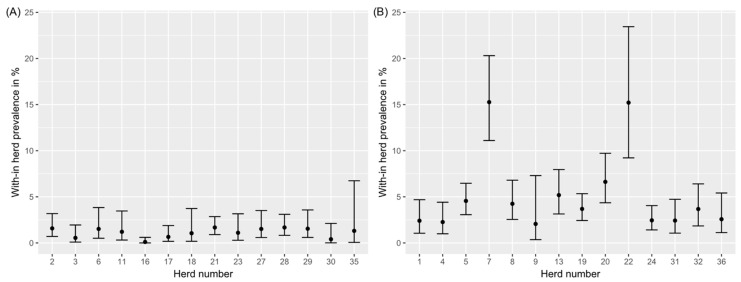
Estimated true within-herd prevalence of animals shedding *Mycobacterium avium* subsp. *paratuberculosis* (MAP) and its 95% confidence interval for each study herd. (**A**) Herds with at least one MAP-positive individual fecal sample and an estimated true prevalence of less than 2% (group 1). (**B**) Herds with at least one MAP-positive individual fecal sample and an estimated true prevalence of at least 2% (group 2).

A true within-herd prevalence of less than 2% was estimated for 14 herds (group 1, Figure 1, left panel). For 14 other herds, the estimated true prevalence was higher (group 2, Figure 1, right panel). In eight herds, no MAP positive result was found and these herds were therefore considered to be MAP non-suspect (group 0).

### 3.2. Environmental Samples

The median interval between on-farm visits was 30 days (interquartile range: 6 days). All herds classified as MAP-infected had at least one MAP-positive environmental sample during the study period, except two herds with MAP-positive individual fecal samples, which had no MAP-positive environmental sample over the entire term of the study. All herds with no MAP-positive individual sample, which were therefore considered to be MAP non-suspect, had no MAP-positive environmental sample. The number of sampling events with a MAP-positive result at herd level can be found in Table A1 in Appendix A.

All environmental samples were evaluable when applying bacterial culture. In contrast, 26 environmental samples and two pools of environmental samples were not evaluable when applying PCR. As each sample was evaluable when applying bacterial culture, the result of this laboratory method could be used when estimating the sensitivity of bacterial culture and fecal real-time PCR taken together. On the grounds of health and safety at work, no liquid manure sample was taken at nine on-farm visits, resulting in three missing manure samples at one farm and six missing manure samples at another farm.

### 3.3. Milk Samples and Milk Pools

In total, 23 out of 28 herds with MAP-positive individual fecal samples had at least one MAP-positive milk pool of size 50. One of the herds not detected as MAP-infected by analyzing milk pools of size 50 had neither a MAP-positive milk pool of size 25, nor a MAP-positive environmental sample. One herd considered to be MAP non-suspect had three MAP-positive pools of size 25, each dedicated to a different month. The same herd had one MAP-positive milk pool of size 50.

For six herds, the milk pools of size 50 dedicated to one month were not evaluable, either because there was too little sample material or due to flakiness of the milk pools. The non-evaluable month was not the same for the six herds.

### 3.4. Statistical Analysis and Model

**Figure 2:**
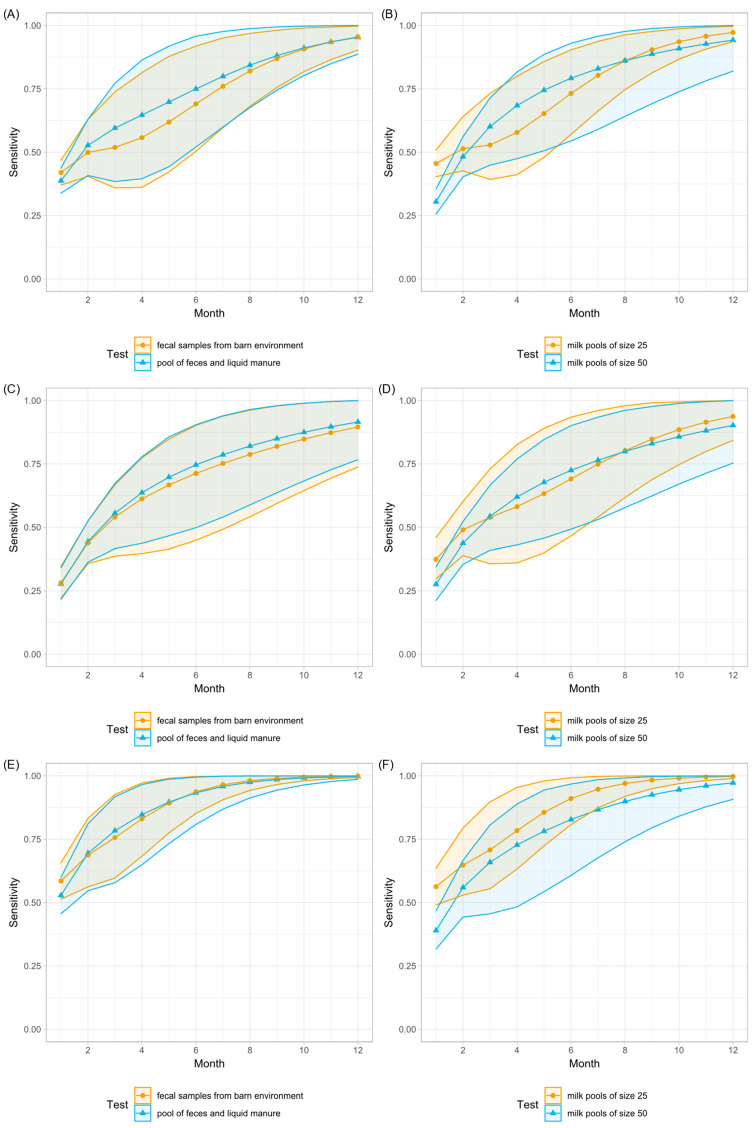
Cumulative sensitivity and 95% CI of environmental samples and pooled milk samples from large low-prevalence dairy herds for the detection of MAP. (**A**,**B**) Model run with the data of all study herds. (**C**,**D**) Model run with the data of all MAP non-suspect herds (group 0) and all MAP-infected herds with a within-herd prevalence of MAP-shedders of less than 2% (group 1). (**E**,**F**) Model run with the data of all MAP non-suspect herds (group 0) and all MAP-infected herds with a within-herd prevalence of MAP-shedders of at least 2% (group 2).

**Figure 3:**
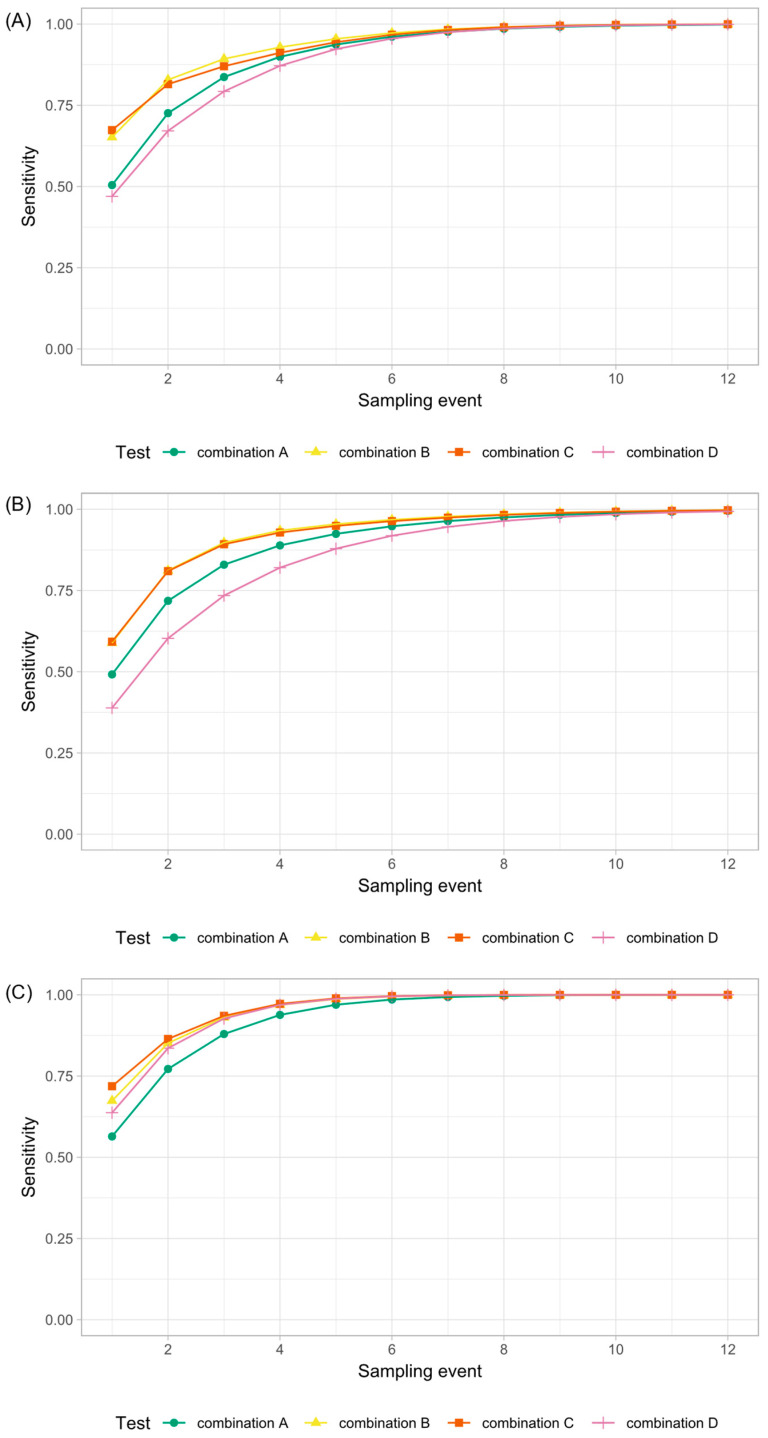
Cumulative sensitivity of different test combinations for the detection of MAP in large low-prevalence dairy herds. (**A**) Model run with the data of all study herds. (**B**) Model run with the data of all MAP non-suspect herds (group 0) and all MAP-infected herds with a within-herd prevalence of MAP-shedders of less than 2% (group 1). (**C**) Model run with the data of all MAP non-suspect herds (group 0) and all MAP-infected herds with a within-herd prevalence of at least 2% (group 2). Combination A: sample sock + milk pools of size 50; Combination B: pool of liquid manure and fecal samples from the barn environment (feces from the milking area, the main alleyway, and the lactating cow floor) + milk pools of size 50; Combination C: fecal samples from the barn environment (milking area, the main alleyway, and the lactating cow floor) + milk pools of size 50; Combination D: sample sock + pool of liquid manure and fecal samples from the barn environment (feces from the milking area, the main alleyway, and the lactating cow floor).

**Figure 4:**
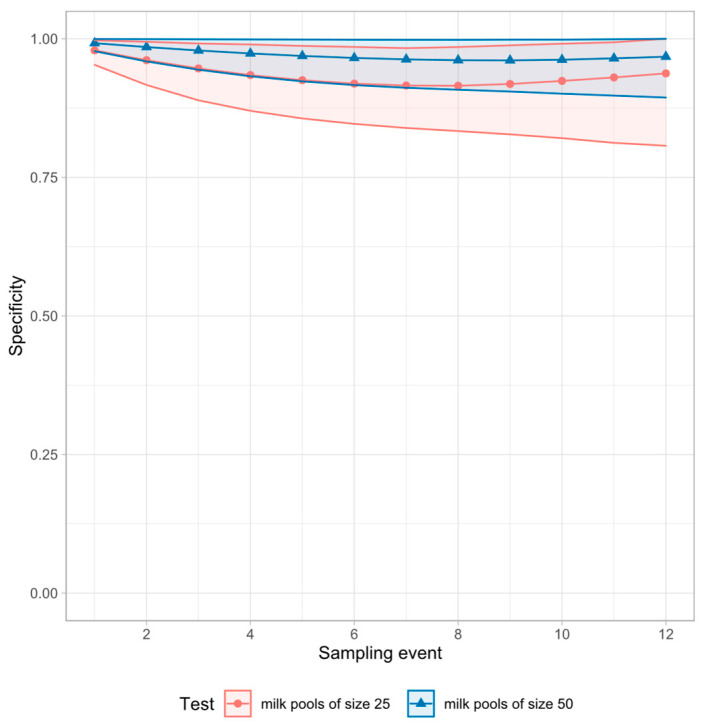
Cumulative specificity and 95% CI of pooled milk samples for the detection of antibodies against MAP. Model run with the data of all herds.

**Figure A1:**
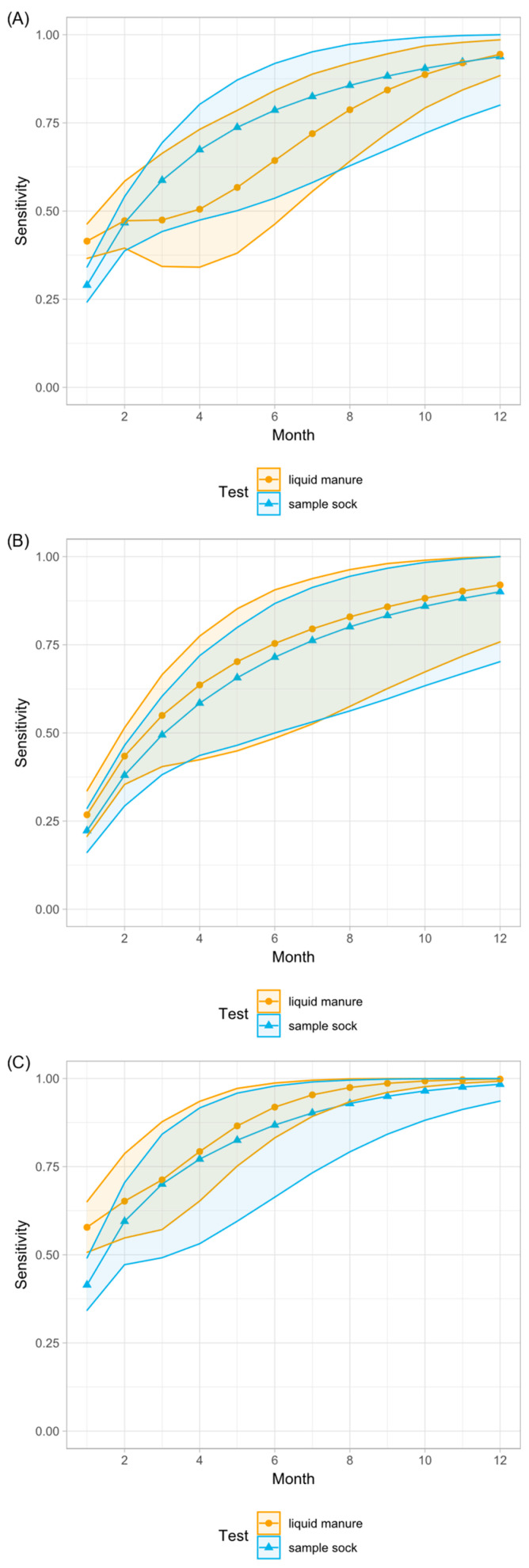
Cumulative sensitivity and 95% credible interval (CI) of sample sock and liquid manure for the detection of *Mycobacterium avium* subsp. *paratuberculosis* (MAP). (**A**) Model run with the data of all study herds. (**B**) Model run with the data of all MAP-free herds (group 0) and all MAP-infected herds with a within-herd prevalence of MAP-shedders of less than 2% (group 1). (**C**) Model run with the data of all MAP-free herds (group 0) and all MAP-infected herds with a within-herd prevalence of at least 2% (group 2).

Regarding only one sampling event, the most sensitive method was analyzing milk pools of size 25 by ELISA (Table 1), but this was the method with the lowest specificity (Table 2). With each repetition of the tests under examination, the cumulative sensitivity increased (Figure 2 and Figure 3), whereas the cumulative specificity tended to decrease, particularly the specificity of milk pools (Figure 4). Detailed results of the cumulative sensitivity of sample socks and liquid manure are given in Figure A1 in Appendix B.

According to the estimates derived from the model, seven sampling events were necessary to detect MAP-infection in herds with a within-herd prevalence of at least 2% with a probability of at least 95% when using fecal samples from the barn environment or liquid manure samples (Figure 2). Using a sample sock, ten sampling events were necessary to achieve the same level of sensitivity in these herds. To achieve a sensitivity of 95% in herds with a prevalence of less than 2%, a combination of different sample types was required (Figure 3).

After assigning a MAP-positive result to all missing or non-evaluable samples, the estimates of the median sensitivity and specificity were very similar to the results based on data with MAP-negative results assigned to all missing or non-evaluable samples. They did not differ by more than 2.7% (absolute), except for the specificity of milk pools of size 50 and test combinations containing these pools. The aberrance in the specificity of milk pools of size 50 increased with more sampling events. The median specificity of milk pools of size 50 was up to 9.9% (absolute) lower when assigning a MAP-positive result to all missing or non-evaluable samples (among them three non-evaluable milk pools of size 50 dedicated to MAP non-suspect herds).

In the fifth model run, the MAP-status of MAP non-suspect herds was considered to be a latent class and herds with at least one MAP-positive individual fecal sample were considered to have a known status (MAP-infected). The results of this model run were compared with the results of the model run at which all study herds were assumed to have a known MAP-status. The comparison revealed that the estimates of sensitivities and specificities were very similar. The maximum difference was 1.7% (absolute).

The visual examination of the MCMC trajectory showed that the chains overlapped well. In addition, the density plots of the chains overlapped well. The potential scale reduction factor was not greater than 1.1 for all mentioned parameters.

### 3.5. Comparison of Laboratory Methods

The sensitivity of the real-time PCR tended to be higher than the sensitivity of bacterial culture (Table 3). This was the case for all types of environmental samples analyzed in the study. Regarding fecal real-time PCR, nine samples were considered to be PCR MAP-negative because of inconclusive PCR results. Applying real-time PCR on liquid manure was significantly more sensitive than the application of bacterial culture, as indicated by the non-overlapping 95% credible intervals (CI). For the combination of both methods, an increase in sensitivity was observed compared with the analysis with only one laboratory method (Table 3).

## 4. Discussion

The objective of our study was to estimate the cumulative sensitivity and specificity of different herd-level surveillance approaches regarding paratuberculosis for up to twelve sampling events in a prospective field study in herds with known disease status and known within-herd prevalence of MAP shedders. The challenge was the low estimated true prevalence of MAP shedders in our study herds, and we aimed to identify the number of repeated samplings needed to achieve a sensitivity of at least 95% in such herds with different combinations of tests. Our study shows that, for the detection of MAP-infected dairy herds with a prevalence of MAP shedders higher than 2%, at least seven repeated samplings of feces from the barn environment are required to achieve a sensitivity of 95%. The detection of herds with a within-herd prevalence of MAP shedders of less than 2% is more challenging, and requires a combination of different samples as well as numerous repetitions. To monitor such herds, a combination of pools of 50 milk samples tested for MAP-specific antibodies, and three fecal samples from the barn environment, tested individually for MAP in parallel, was identified as the most sensitive out of the combinations under examination. Similarly, the combination of testing a pool of these environmental samples and liquid manure, in addition to the milk pools, also showed a high sensitivity.

In comparison with monitoring strategies based on individual testing, the advantages of monitoring herds by environmental sampling and pooling milk samples taken in the context of monthly dairy herd improvement tests are low costs, as well as easy and time-saving sample collection, as no additional handling of individual animals is necessary [32,48].

In our study, the environmental samples were taken by different veterinarians. As several studies have shown that the results of samples collected by different persons are comparable [58,59,60], we do not assume that the different investigators led to bias in our results.

With regard to the model, we assumed that the status of each study herd remained constant over the time of our study. In order to reduce a potential misclassification bias caused by variation in the disease status, we limited the study period to approximately one year. On the one hand, the assumption of a temporally static herd status is supported by the fact that the within-herd prevalence changes slowly, even if preventive measures are applied [19,61]. On the other hand, it has to be considered that the within-herd prevalence of our MAP-infected study herds might have declined because all study herds participated in the voluntary Thuringian paratuberculosis control program, which stipulates the culling of animals within one month after the receipt of a MAP-positive result of their individual fecal sample [42]. The culling of animals that have tested MAP-positive reduces the within-herd prevalence [62,63]. The within-herd prevalence, in turn, has an effect on the sensitivity of environmental sampling [32,33,34,35] and milk testing [28]. A declining prevalence during the course of the study may have led to an underestimation of sensitivity in our study.

The low within-herd prevalence in our MAP-infected study herds hampers the comparison of our results with the results of other studies because their study herds had a higher within-herd prevalence, which affects estimates of sensitivity [29]. Other factors at herd and at animal level potentially influence estimates of sensitivity and specificity and therefore the comparability between studies. At animal level, the stage of the disease should be considered [64]. At herd level, an example of such a factor is difference between study populations [65]. Additionally, generalization and comparability are limited by the number of our study herds and the applied laboratory methods. A Bayesian modelling, based on data from the Alberta Johne’s Disease Initiative, found that eight sampling events with two environmental samples per sampling event are necessary to achieve a cumulative sensitivity of at least 90% with 95% probability [41]. It is worth mentioning that the data in that study were available only for three sampling events, and that the herd status was unknown. As is known from other studies, the estimated within-herd prevalence of MAP-seropositive cows in that Canadian region is 7–9% [66,67]. The model-based predications made on the basis of those data are comparable to our results if the higher within-herd prevalence in the Canadian study population is taken into account. Another Canadian study estimated the sensitivity to be 93% for six annual environmental samples collected in three consecutive years [68], which is similar as well. In another study conducted in herds with a median apparent within-herd MAP shedder prevalence of approximately 7%, six sampling events with six samples taken at each sampling event were necessary to reach a sensitivity of 90% [33]. This result is similar to our finding that pooled fecal samples from the barn environment achieve a sensitivity of 95% after seven sampling events in herds with a within-herd prevalence of at least 2%. When discussing these results, it is important to keep in mind that even the testing of individual fecal samples provides a limited sensitivity. This also applies to testing individual milk samples for MAP-specific antibodies, even to a larger extent.

A major advantage of our study design is the availability of a reliable reference test, which allows the classification of the study herds (MAP-infected or MAP non-suspect) with high certainty. Furthermore, we were able to apply a real-time PCR for direct detection of MAP without a previous cultivation step on the environmental samples. Although application of PCR is preferred by most laboratories for MAP detection in fecal samples, a review of herd-level diagnostics for MAP revealed knowledge gaps concerning herd sensitivity of environmental samples analyzed by PCR [69]. Compared to previous studies, the combined testing of environmental samples for MAP and pooled milk samples for MAP-specific antibodies, as well as the high number of sampling repetitions, was a strength of our study.

As we tested the same herds several times using the same test methods, it could not be assumed that the test results were independent. A correlation between repeated tests was shown for the detection of MAP-specific antibodies in milk by ELISA [31] and for environmental samples [41]. In addition to the correlation at the temporal level, the correlation between different tests or their combinations had to be taken into account, because it is likely that the different test methods used in our study were correlated, as they measured similar biological phenomena [70]. Our study includes tests that concern different biological phenomena, namely the fecal excretion of MAP as detected by testing environmental samples and the humoral immune reaction which is reflected by MAP-specific antibodies detected by ELISA in milk samples.

The hierarchical structure of our model and the resulting shrinkage could explain why the specificity of repeated environmental sampling was estimated to be smaller than 100%, even though none of the environmental samples from MAP non-suspect study herds showed any MAP-positive result. Shrinkage arises because the estimates of low-level parameters are influenced by higher-level parameters [71]. It seems reasonable to suppose that the specificity of environmental sampling is higher than estimated by our model, as we had no false-positive environmental sample in our study.

In contrast, we assume that three MAP-positive milk pools of size 25 were false positive. These MAP-positive pools were dedicated to one herd, but to three different test days (February, March, December). The herd was considered to be MAP non-suspect as it has been certified as MAP non-suspect for a long time and was adequately monitored. The individual MAP status of all cows whose individual samples contributed to the MAP-positive milk pools was verified. All cows had a MAP-negative individual fecal sample in 2020 or 2021, except for three cows whose individual milk samples contributed to the MAP-positive milk pool in December, but whose feces could not be sampled because the animals were removed from the herd before a follow-up examination could be carried out. Therefore, we have to suppose that at least the pools of February and March were false-positive. It is acknowledged that, especially in low-prevalence herds, the predictive value of ELISA-positive results is low [72].

The model proposed by Wang and Hanson [53] was used for several reasons: firstly, the model is developed for a setting that matches our study design, with serval binary tests administered repeatedly on the same units: in our case, herds. Secondly, an advantage of the model is that it considers temporal covariances as well as covariances between different tests. Thirdly, the model can be applied in settings without a gold standard. Although we used the results of individual fecal culture or PCR as reference tests at herd level, it must be noted that this is not a perfect test. For ante mortem diagnosis of paratuberculosis, a perfect gold standard test is still lacking, and latent class models are very useful for dealing with this lack of true disease status. In our case, we suppose that the latent class model may have difficulties in dealing with the way that many of our study herds had a very low within-herd prevalence and therefore numerous false-negative results. Our suspicion is based on the following two facts. Firstly, the between-herd prevalence was underestimated by the latent class model when compared with the results of our reference test at herd level. Secondly, the specificity of environmental samples tested with culture and PCR was underestimated when taking into account the high specificity of fecal culture and PCR. For this reason, we decided to use the model adapted for cases where the true disease status is known. Even if individual fecal culture is not a perfect gold standard, it is often considered as a reference test [69], and we presume that individual fecal culture or PCR was able to correctly classify our MAP-infected study herds. In contrast, the classification of MAP non-suspect herds is more difficult, and is associated with a higher degree of uncertainty. On the one hand, the sensitivity of our reference test is not perfect, which reduces certainty. On the other hand, most of the MAP-negative herds had a long history of being MAP-negative, and they are certified as MAP non-suspect and monitored adequately. This fact enhances the certainty of the correct classification as MAP non-suspect. Nevertheless, we could not be absolutely sure about the MAP non-suspect status of our respective study herds. That is why we conducted a model run where the status of these herds was considered to be a latent class. This approach can be very useful for other field studies or practical application, because it tackles the common problem of unknown disease status of herds that are either not tested or have had only MAP-negative results, which can be due to poor sensitivity or to the absence of the pathogen. The authors of a recent review of the characteristics of herd level tests for MAP stated that latent class models should be a standard approach to enhance test evaluations for paratuberculosis [69]. There exists more than one reference test for MAP, and latent class models can generate more generalizable outputs [69]. An evaluation of whether latent class models are suitable for situations with very low prevalence and many presumably false-negative results is beyond the scope of this study, but should be evaluated in simulation studies (see, e.g., [73,74]).

The estimates of herd sensitivity and herd specificity as key test performance parameters at herd level [69] provide useful information for evaluating our approach for monitoring large dairy herds certified as MAP non-suspect with the aim to detect a (re-)entry of the causative organism early. The estimates of sensitivity can be used to measure the quality of the monitoring system, but can also answer the extended question regarding the herd-level confidence of freedom from disease (“herd assurance”), given a fixed number of sampling events with negative results [75]. Moreover, the estimates of sensitivity and specificity can help decision makers, and they can serve as inputs for simulation modelling [64].

## 5. Conclusions

Repeated testing of large dairy herds with a combination of environmental samples and pooled milk samples is a sensitive method for detecting herds with a low within-herd prevalence of MAP shedders. This is crucial for the detection of a (re-)entry of the infectious agent, and qualifies this approach for surveillance purposes in the framework of control programs where MAP non-suspect dairy herds have to be monitored for maintenance of status. If a within-herd prevalence of MAP shedders of more than 2% is to be detected, the use of either environmental samples or pooled milk samples is recommendable. The sensitivity in large herds with an even lower within-herd prevalence is not good enough to detect these herds with a high certainty by using either environmental samples or pooled milk samples, even if the sampling is repeated twelve times. Therefore, a combination of several different sample types has to be collected and analyzed repeatedly.

## Figures and Tables

**Table 1:** Posterior mean, median, and 95% credible interval (CI) of the sensitivity regarding the detection of MAP at herd level (one sampling event) in large low-prevalence dairy herds.

Sample Type	Laboratory Method	Mean	Median	95% CI
Sample sock	PCR + culture ^b^	0.290	0.290	0.242–0.341
Liquid manure	PCR + culture ^b^	0.415	0.415	0.366–0.463
Pool of fecal samples from the barn Environment ^a^ and liquid manure	PCR + culture ^b^	0.387	0.387	0.338–0.437
Fecal samples from the barn environment ^a^	PCR + culture ^b^	0.420	0.420	0.370–0.468
Milk pools of size 25	ELISA	0.456	0.456	0.404–0.508
Milk pools of size 50	ELISA	0.305	0.305	0.256–0.355

^a^ Feces from the milking area, the main alleyway, and the lactating cow floor. ^b^ A sample was considered to be MAP-positive if tested positively by bacterial culture or fecal PCR or both, otherwise the sample was considered to be MAP-negative.

**Table 2:** Posterior mean, median, and 95% CI of the specificity regarding the detection of MAP at herd level (one sampling event) in large low-prevalence dairy herds.

Sample Type	Laboratory Method	Mean	Median	95% CI
Sample sock	PCR + culture ^b^	0.993	0.995	0.981–1.00
Liquid manure	PCR + culture ^b^	0.991	0.993	0.976–1.00
Pool of fecal samples from the barn Environment ^a^ and liquid manure	PCR + culture ^b^	0.994	0.995	0.982–1.00
Fecal samples from the barn environment ^a^	PCR + culture ^b^	0.992	0.994	0.978–1.00
Milk pools of size 25	ELISA	0.977	0.979	0.953–0.997
Milk pools of size 50	ELISA	0.991	0.992	0.978–1.00

^a^ Feces from the milking area, the main alleyway, and the lactating cow floor. ^b^ A sample was considered to be MAP-positive if tested positively by bacterial culture or fecal PCR or both, otherwise the sample was considered to be MAP-negative.

**Table 3:** Posterior median and 95% CI of the sensitivity of environmental samples from large low-prevalence dairy herds, analyzed by bacterial culture or real-time PCR or both laboratory methods, regarding the detection of MAP at herd level (one sampling event).

	Only Culture		Only PCR		PCR + Culture ^b^	
Sample Type	Median	95% CI	Median	95% CI	Median	95% CI
Sample sock	0.203	0.163–0.246	0.237	0.191–0.284	0.290	0.242–0.341
Liquid manure	0.205	0.163–0.247	0.376	0.328–0.428	0.415	0.366–0.463
Pool of fecal samples from the barn environment ^a^ and liquid manure	0.248	0.202–0.295	0.335	0.286–0.384	0.387	0.338–0.437
Fecal samples from the barn environment ^a^	0.343	0.292–0.398	0.348	0.299–0.397	0.420	0.370–0.468

^a^ Feces from the milking area, the main alleyway, and the lactating cow floor. ^b^ A sample was considered to be MAP-positive if the bacterial culture or the fecal PCR or both produced a positive result, otherwise the sample was considered to be MAP-negative.

## Data Availability

The data presented in this study are available in Table A1.

## Appendix A

**Table A1:** Number of sampling events per herd with a *Mycobacterium avium* subsp. *paratuberculosis* (MAP)-positive result at herd level.

		Number of Sampling Events with a MAP-Positive Result at Herd Level					
Herd Number	Estimated True within-Herd Prevalence of MAP-Shedders	Sample Sock	Liquid Manure	Fecal Samples from the Barn Environment	Pool of Feces ^a^ and Liquid Manure	Milk Pools of Size 25	Milk Pools of Size 50
1	2.41	0	0	1	1	2	2
2	1.58	2	1	2	1	2	1 ^d^
3	0.55	0	1	0	0	0	0
4	2.27	6	8	11	8	7	5
5	4.56	6	10	9	7	12	6
6	1.52	1	2 ^b^	4	5	3	2 ^d^
7	15.27	12	12	12	12	12	11
8	4.25	2	12	8	6	8	3
9	2.06	1	5	3	2	9	4
10	0.00	0	0	0	0	0	0
11	1.21	0	0	0	0	2	1
12	0.00	0	0	0	0	0	0
13	5.19	0	0	4	2	0	0
14	0.00	0	0	0	0	0	0 ^d^
15	0.00	0	0	0	0	0	0
16	0.11	0	2	0	1	4	2
17	0.66	0	0	0	0	0	0
18	1.06	0	1	1	1	2	0 ^d^
19	3.69	4	5 ^c^	7	8	6	1
20	6.63	8	8	7	7	8	8
21	1.68	0	1	1	0	3	2
22	15.21	7	11	11	9	9	4
23	1.10	3	2	4	4	8	5
24	2.45	7	9	9	7	10	6 ^d^
25	0.00	0	0	0	0	0	0 ^d^
26	0.00	0	0	0	0	3	1
27	1.52	2	2	6	3	5	2
28	1.68	1	2	1	1	9	4
29	1.55	8	9	9	10	12	12
30	0.40	0	1	2	1	1	1
31	2.43	4	6	8	7	2	1
32	3.68	4	5	8	5	8	4
33	0.00	0	0	0	0	0	0
34	0.00	0	0	0	0	0	0
35	1.31	7	12	9	10	9	4
36	2.57	0	4	0	3	0	0

^a^ Feces from the milking area, the main alleyway, and the lactating cow floor. ^b^ Manure samples were collected for only six months. On health and safety grounds, no manure samples were collected for six months. ^c^ Manure samples were collected for only nine months. On health and safety grounds, no manure samples were collected for three months. ^d^ Milk pools dedicated to one month were not evaluable.
